# Pretreatment with statins improves early outcome in patients with first-ever ischaemic stroke: a pleiotropic effect of statins or a beneficial effect of hypercholesterolemia?

**DOI:** 10.1186/1471-2377-10-47

**Published:** 2010-06-18

**Authors:** Adrià Arboix, Luis García-Eroles, Montserrat Oliveres, Cecília Targa, Miquel Balcells, Joan Massons

**Affiliations:** 1 Unit of Cerebrovascular Diseases, Service of Neurology, Hospital Universitari del Sagrat Cor, Universitat de Barcelona, Barcelona, Spain; 2 CIBER de Enfermedades Respiratorias (CB06/06), Instituto Carlos III, Madrid, Spain; 3 Unit of Organization, Planning and Information Systems, Consorci Sanitari del Maresme, Barcelona, Spain

## Abstract

**Background:**

Data from different studies suggest a favourable association between pretreatment with statins or hypercholesterolemia and outcome after ischaemic stroke. We examined whether there were differences in in-hospital mortality according to the presence or absence of statin therapy in a large population of first-ever ischaemic stroke patients and assessed the influence of statins upon early death and spontaneous neurological recovery.

**Methods:**

In 2,082 consecutive patients with first-ever ischaemic stroke collected from a prospective hospital-based stroke registry during a period of 19 years (1986-2004), statin use or hypercholesterolemia before stroke was documented in 381 patients. On the other hand, favourable outcome defined as grades 0-2 in the modified Rankin scale was recorded in 382 patients.

**Results:**

Early outcome was better in the presence of statin therapy or hypercholesterolemia (cholesterol levels were not measured) with significant differences between the groups with and without pretreatment with statins in in-hospital mortality (6% *vs *13.3%, *P *= 0.001) and symptom-free (22% *vs *17.5%, *P *= 0.025) and severe functional limitation (6.6% *vs *11.5%, *P *= 0.002) at hospital discharge, as well as lower rates of infectious respiratory complications during hospitalization. In the logistic regression model, statin therapy was the only variable inversely associated with in-hospital death (odds ratio 0.57) and directly associated with favourable outcome (odds ratio 1.32).

**Conclusions:**

Use of statins or hypercholesterolemia before first-ever ischaemic stroke was associated with better early outcome with a reduced mortality during hospitalization and neurological disability at hospital discharge. However, statin therapy may increase the risk of intracerebral haemorrhage, particularly in the setting of thrombolysis.

## Background

Recent data have shown that lipid-lowering treatment using HMG-CoA reductase inhibitors (statins) is effective in primary and secondary stroke prevention in high risk patients for vascular disease [1,2]. Furthermore, statin therapy discontinuation has been associated to unfavourable stroke outcome [3,4]. Statins induce several potential neuroprotective effects including an increase in cerebral perfusion, activation of survival signals, synthesis of heat shock protein 27, and increases in angiogenesis and neurogenesis [5]. Statins may protect the brain when administered shortly after injury but also afford protective effects in patients taking statins for hypercholesterolemia before the injury [4]. For patients with acute ischaemic stroke premorbid statin use was associated with smaller lesion volume and better functional outcome [1,6,7] and high levels of total cholesterol were associated with a increased rate of recovery from disability in basic activities of daily living among hospitalized older adults [8] and decreased risk of physical disability after stroke [9].

To further contribute to determine the effects of statins on early outcome in patients with first-ever ischaemic stroke, data of 2,082 consecutive ischaemic stroke patients included in a prospective stroke registry during a 19-year period were analysed. The objectives of the study were as follows: *a*) to compare early outcome in the subsets of acute ischaemic stroke patients with and without statin use before stroke and *b*) to assess the effect of statin therapy on in-hospital mortality and spontaneous early neurological recovery.

## Methods

Since January 1986, the Hospital of Sagrat Cor (an acute care 350-bed teaching hospital in the city of Barcelona serving a population of ~250,000 people) has had an ongoing hospital-based stroke registry. Data from first-ever stroke patients are entered following a standardized protocol with 186 items regarding demographics, anamnestic data (vascular risk factors, underlying illnesses, concomitant treatments), clinical features, laboratory and neuroimaging data, complications and outcome. Data included in the registry up to December 2004, the particular time at which 3,808 patients had been entered in the database were reviewed. Classification of subtypes of stroke and definition of cardiovascular risk factors were those used by our group in previous studies [10]. For the purpose of this study, the group of 2,082 consecutive patients with first-ever ischemic stroke was selected. Prior to conducting the study, approval was obtained from Ethical Committee on Clinical Research of the hospital.

All patients were admitted to the hospital within 48 hours of onset of symptoms. On admission, demographic characteristics; salient features of clinical and neurological examination and results of laboratory tests (blood cell count, biochemical profile, serum electrolytes, urinalysis); chest radiography; and twelve-lead electrocardiography; and brain CT and/or MRI were recorded. Other investigations, such as angio-MRI, echo-Doppler of the supra-aortic trunks, arterial digital subtraction angiography, B-mode echocardiography and lumbar puncture were performed at the discretion of the neurologist in charge.

Degree of clinical disability at discharge from the hospital was evaluated according to the modified Rankin scale (mRS), a 6-item functional disability scale (grade 0 = no symptoms at all; grade 1 = symptoms but able to carry out all usual activities; grade 2 = slight disability but able to carry out activities without assistance; grade 3 = moderate disability, requiring some help but able to walk without assistance; grade 4 = moderately severe disability; and grade 5 = severe disability: bedridden, incontinent, and requiring constant nursing care and attention). The mRS was obtained prospectively. The outcome of patients was classified as favourable outcome (spontaneous neurological recovery or minimal stroke associated disability at discharge (mRS grades 0-2), and unfavourable outcome (not improved, mRS grades 3-5 or in-hospital death). The cut-off point of the Rankin scale was defined before the study. Causes of death were analysed according to criteria of Silver et al. [11]. Moreover, outcome variables included cardiac events (acute myocardial infarction, heart failure, or tachyarrhythmia); respiratory events (pulmonary embolism, atelectasis, or lower respiratory tract infection); infectious complications, neurological complications (progressive stroke, early seizures or early stroke recurrence) and urinary complications.

### Statistical analysis

Data from patients with and without statin use before stroke were analysed using the Student's *t *test or the Mann-Whitney *U *test for the comparison of continuous variables and the chi-square (χ^2^) test (with Yates correction when necessary) for the comparison of categorical variables. In addition, data from patients with and without favourable outcome (mRS grades 0-2) were compared with the Student's *t *test, χ^2 ^test and the analysis of variance (ANOVA) when appropriate. Statistical significance for inclusion in the multivariate analysis was set at *P *< 0.02. Variables related to either in-hospital death or favourable outcome in the univariate analyses plus sex and age were subjected to multivariate analysis with a logistic regression procedure. All variables selected were included in the initial model. Criteria for variables to remain in the model were based on the statistical significance of the Wald's test (*P *< 0.05) and on the comparison of the estimated coefficient of each variable with the coefficient of the model containing only this variable. Variables that did not contribute to the model according to these criteria were removed and the model readjusted. The new model after removing a variable was compared with the former model with the likelihood ratio test. Age was used in multivariate analysis as a continuous variable with a constant odds ratio for each year. In the two models, in-hospital mortality (coded as alive = 0 and death = 1) and favourable outcome (coded as absence = 0, presence = 1) were the dependent variables. Odds ratio (OR) and 95% confidence intervals (CI) were calculated from the beta coefficients and standard errors. The hypothesis that the logistic regression model adequately fitted the date was tested by means of the goodness of fit χ^2 ^test [12]. The SPSS-PC+ and BMDP computer programmes were use for statistical analyses.

## Results

A total of 381 patients were receiving statin treatment at the time of brain infarction. These patients accounted for 18.3% of all cases of first-ever ischaemic stroke included in the stroke registry. Statin-treated patients as compared with the untreated group were significantly younger and the proportion of patients aged ≥ 85 years of age was also significantly lower. When differences in outcome variables between statin-treated and untreated patients were analysed, early outcome was better in the statin group (Table 1), with a significantly higher percentage of favourable outcome (mRS grade 0) at the time of hospital discharge, a significantly lower percentage of patients with severe functional limitation at hospital discharge as well as lower rates of infectious complications and respiratory events during hospitalisation. The proportion of patients with neurological complications was also lower in the statin-treated group and almost reached statistical significance. In-hospital mortality was 13.3% (*n *= 226) among untreated patients and 6.0% (*n *= 26) among statin-treated patients. Causes of death were cerebral herniation in 7 patients, sepsis in 6, myocardial infarction in 4, sudden death in 2, lower respiratory tract infection in 2 and unknown cause in 2. The median (25th-75th percentile) length of hospital stay was 10 (8-18) days in patients treated with statins as compared with 12 (8-20) days in untreated patients (*P *= 0.001).

**Table 1:** Results of univariate analysis: differences in outcome variables between first-ever ischaemic stroke patients with and without pretreatment with statins

Data	Statin use		*P *value
		
	Yes (*n *= 381)	No (*n *= 1,701)	
Male sex	194 (50.9)	793 (46.6)	0.072

Age, years, mean (SD)	71.8 (11.1)	75.7 (12.3)	0.000

Age, years ≥ 85 years	44 (11.5)	380 (22.3)	0.000
Cardiac events	17 (4.5)	78 (4.6)	0.523

Respiratory events	21 (5.5)	162 (9.5)	0.006

Infectious complications	37 (9.7)	223 (13.1)	0.039

Neurological complications	28 (7.3)	173 (10.2)	0.053

Favourable outcome, mRS grades 0-2	84 (22)	298 (17.5)	0.025

Unfavourable outcome, mRS grades 3-5	25 (6.6)	196 (11.5)	0.002

In-hospital mortality	23 (6.0)	226 (13.3)	0.000

mRS: modified Rankin scale.

Percentages in parenthesis unless otherwise stated.

Of the total series of 2,028 patients with first-ever ischaemic stroke, favourable outcome was recorded in 382 patients (18.8%). On the other hand, the overall in-hospital mortality rate was 11.9% (*n *= 249). Results of univariate analysis in ischemic stroke patients according to vital status (alive, death) and presence or absence of favourable outcome (mRS grade 0-2) at hospital discharge are shown in Table 2. Patients with unfavourable outcome (death or mRS grades 3-5) compared with those with favourable outcome (early neurological recovery or minimal stroke associated disability at discharge) were significantly older and had more frequently ischaemic heart disease, atrial fibrillation, congestive heart failure and chronic obstructive pulmonary disease (COPD) as vascular risk factors. With regard to clinical variables, sudden onset, early seizures, nausea/vomiting, altered consciousness, limb weakness, sensory symptoms and hemianopia were also significantly more frequent among patients with unfavourable outcome. In the statins pretreatment group, not only a significantly higher percentage of patients were discharged alive from the hospital (19.5% *vs *9.2%) but also there was a higher percentage of patients with favourable outcome (22% *vs *17.5%) than in the untreated group (Table 2). As shown in Table 3, the frequency of favourable outcome and in-hospital mortality in relation to the use of statins when the study period was divided into three periods (1986-1992; 1993-1998; 1999-2004) did not show significant differences, although a decreasing trend in the mortality rate was observed.

**Table 2:** Results of univariate analysis: differences in demographics, vascular risk factors and clinical features in acute ischaemic stroke patients according to outcome

Data	Vital status at discharge			Favourable outcome (mRS grades 0-2)		
	
	Alive	Death	*P *value	Yes	No	*P *value
No. patients	1833	249		382	1700	

Male sex	882 (48.1)	105 (42.2)	0.045	195 (51.0)	792 (46.6)	0.064

Age, years, mean (SD)	80.7 (8.9)	74.2(12.4)	0.000	74.6 (10.9)	75 (12.5)	0.014

Age, years ≥ 85 years	330 (18)	94 (37.8)	0.000	56 (14.7)	368 (21.6)	0.001

Vascular risk factors						

Hypertension	997 (54.4)	123 (49.4)	0.077	200 (52.4)	928 (54.6)	0.244

Diabetes mellitus	421 (23)	50 (20.1)	0.174	93 (24.3)	78 (4.6)	0.142

Valvular heart disease	111 (6.1)	19 (7.6)	0.202	28 (7.3)	102 (6)	0.195

Ischaemic heart disease	168 (9.2)	57 (22.9)	0.001	50 (13.1)	269 (15.8)	0.102

Atrial fibrillation	481 (26.2)	128 (51.4)	0.000	94 (24.6)	515 (30.3)	0.015

Congestive heart failure	76 (4.1)	32 (12.9)	0.000	9 (2.4)	99 (5.8)	0.002

Transient ischaemic attack	204 (11.1)	33 (13.2)	0.187	48 (12.6)	189 (11.1)	0.235

Peripheral arterial disease	127 (6.9)	19 (7.6)	0.382	26 (6.8)	120 (7.1)	0.483

Chronic pulmonary disease	132 (7.2)	34 (13.7)	0.001	21 (5.5)	145 (8.5)	0.027

Obesity (BMI > 32.5 kg/m^2^)	97 (5.3)	6 (2.4)	0.060	24 (6.3)	79 (4.6)	0.116

Alcohol abuse (> 80 g/day)	47 (2.6)	9 (3.6)	0.219	7 (1.8)	49 (2.9)	0.166

Smoking (> 20 cigarettes/day)	193 (10.5)	14 (5.6)	0.013	32 (8.4)	175 (10.3)	0.149

Clinical features						

Sudden onset	910 (49.6)	144 (57.8)	0.009	187 (49)	867 (51)	0.253

Headache	242 (13.2)	17 (6.8)	0.002	47 (12.3)	212 (12.5)	0.504

Dizziness	71 (3.9)	8 (3.2)	0.383	18 (4.7)	66 (3.9)	0.395

Early seizures	23 (1.2)	14 (5.6)	0.000	4 (1.0)	33 (1.9)	0.163

Nausea, vomiting	132 (7.2)	33 (13.2)	0.001	31 (8.1)	134 (7.9)	0.474

Speech disturbances	925 (50.5)	147 (59.0)	0.007	200 (52.4)	872 (51.3)	0.375

Altered consciousness	189 (10.3)	149 (59.8)	0.000	24 (6.3)	314 (18.5)	0.000

Limb weakness	1334 (72.8)	232 (93.2)	0.000	259 (67.8)	1307 (76.9)	0.000

Sensory symptoms	672 (36.7)	137 (55.0)	0.000	109 (28.5)	700 (41.2)	0.000

Hemianopia	293 (16)	76 (30.5)	0.000	30 (7.9)	339 (19.9)	0.000

Ataxia	122 (6.7)	7 (2.8)	0.008	25 (6.5)	104 (6.1)	0.414

Cranial nerve palsy	85 (4.6)	16 (6.4)	0.122	14 (3.7)	87 (5.1)	0.143

Statin use before stroke	356 (19.5)	23 (9.2)	0.000	84 (22)	297 (17.5)	0.025

Hospital stay, days, median (25th-75th interquartile range)	12 (9-20)	10 (4.7-23)	0.000	10 (8-14)	13 (8-22)	0.000

Percentages in parenthesis unless otherwise stated. Percentages are calculated for the total number in each column.

**Table 3:** Frequency of favourable outcome and in-hospital mortality in statin users during the study period

Outcome	1986-1992* (n = 146)	1993-1998 (n = 107)	1999-2004 (n = 128)	*P *value*
Favourable outcome (n = 84)	33 (22.6)	24 (22.4)	27 (21.1)	0,950

In-hospital mortality (n = 23)	18 (8.2)	6 (5.6)	5 (3.9)	0.319

*Statin use or hypercholesterolemia.

In the logistic regression model statin use before ischaemic stroke was the only variable inversely associated with in-hospital death (OR = 0.57) and directly associated with favourable outcome (OR = 1.32) (Table 4).

**Table 4:** Results of multivariate analyses

Variable	β	SE (β)	Odds ratio (95% CI)	*P *value
In-hospital mortality*				

Atrial fibrillation	0.804	0.143	2.23 (1.69-2.96)	0.000

Congestive heart failure	0.728	0.235	2.07 (1.31-2.28)	0.002

Chronic obstructive pulmonary disease	0.539	0.216	1.71 (1.12-2.62)	0.013

Age	0.047	0.008	1.05 (1.03-1.06)	0.000

Statin use before stroke	-0.568	0.234	0.57 (0.36-0.89)	0.002

Favourable outcome (mRS grades 0-2)^†^				

Statin use before stroke	0.274	0.139	1.32 (1.01-1.73)	0.049

Congestive heart failure	-0.926	0.353	0.40 (0.20-0.79)	0.009

* β = -6.029, SE (β) = 0.649, goodness of fit χ^2 ^= 8.929, d.f. = 8, *P *= 0.348.

^† ^β = -1.511, SE (β) = 0.065, goodness of fit χ^2 ^= 0.041, d.f. = 1, *P *= 0.839.

## Discussion

The present study adds evidence of the good functional outcome at discharge from the hospital in patients who were taking statins at the time of the ischaemic stroke onset. The hypothesis that a statin therapy favours a better early outcome is supported by a significantly lower in-hospital mortality rate and a higher percentage of patients with symptom-free at hospital discharge or minimal stroke associated disability at discharge in patients with statins use than in those without statins pretreatment.

These findings obtained in a large population of over 2,082 patients with first-ever acute ischaemic stroke are consistent with several studies that reported an association between premorbid statin use and better clinical stroke outcome [13-16]. In a recent study of our group in addition to the high frequency of cognitive impairment in patients with multiple recurrent lacunes, an interesting finding was the protective role of hyperlipidemia and statin therapy [17]. These data agree with those of a recent clinical study that provide evidence that previous treatment with statins is an independent factor associated with good outcome in patients with ischaemic stroke [18]. Atherothrombotic and small vessel strokes showed the greatest benefit [17]. In the study of Yoon et al. [19], statin pretreatment was significantly associated with an improved functional outcome, and in the series of Reeves et al., in-hospital mortality was 2.3% among statin users compared to 6.6% in the subjects not treated with statins [20]. In addition, in a meta-analysis totalled 70,388 participants without established cardiovascular disease but with cardiovascular risk factors, statin use was associated with significantly improved survival and large reductions in the risk of major cardiovascular events [3].

Statin drugs improve the outcome of ischaemic stroke patients through different mechanisms, including better cerebral collateral supply, a direct neuroprotective effect, plaque stabilizing effects, and induction of angiogenesis, neurogenesis and synaptogenesis [21-29]. The Stroke Prevention with Aggressive Reduction in Cholesterol (SPARCL) study found that the use of high-dose atorvastatin as compared to placebo in accurately selected patients who had a stroke or TIA was associated with a non-significant 13% risk reduction of non-fatal stroke during a 5-year follow-up without improving survival [30].

Alternative explanations focusing on potentially neuroprotective properties of cholesterol should be considered. Presumably, baseline cholesterol levels of the statin-treated patients in this study were higher than those of non-statin treated patients. Unfortunately, cholesterol levels were not considered in the study. Cholesterol is essential for normal cell membrane fluidity and it has been speculated that high cholesterol concentrations may have neuroprotective effect through increasing gamma-glutamyltransferase. This enzyme plays a role in amino acid uptake and transport and could reduce the neurotoxic effects of excitotoxic amino acids [31]. The proven action of cholesterol as a buffer neutralizing free radicals an also provide antioxidant protection [32,33]. This action might limit the extent of cerebral infarction and increasing the cellular recuperation capacity. In the experimental model of myocardial ischemia, mice fed the high-cholesterol diet for 12 weeks showed a significantly lower area of myocardial infarction compared with mice fed a normal diet [33]. Similarly, enrichment of cardiomyocites cultures with free cholesterol resulted in an increased tolerance to anoxia [34]. It has been shown that elevated cholesterol concentrations were associated with improved short-term health outcome after acute stroke of any type [35], short-term mortality following ischaemic stroke is higher in older patients with low total cholesterol levels independent of a large number of factors [36] and elevated levels of cholesterol were associated with increased rate of recovery from disability in basic activities of daily living among acutely ill hospitalised older patients [8]. Alternatively, we cannot exclude that elevated cholesterol may simply represent an indicator of good nutritional status. In line with this hypothesis, previous observations have identified strong correlations between specific clinical markers of nutritional status (in particular serum proteins) and the risk of subsequent in-hospital adverse events [37].

Accordingly, hyperlipidemia may be considered, on the one hand, a cardiovascular risk factor, but on the other hand, and paradoxically, through a mechanism of neuroprotection of the brain would be related to a lower neurological deficit and decreased mortality in patients with acute cerebral ischaemia. In this respect, when prescribing pharmacological treatment, an excessive reduction of total cholesterol levels may be avoided in order to control the stroke risk factor without affecting the neuroprotection mechanism of hypercholesterolemia. On the basis of this hypothesis, a prospective randomised trial with statins assessing the relationship between functional outcome and mortality with different blood lipid levels in the acute phase of cerebral ischaemia would be necessary.

The present findings should be interpreted taking into account some limitations of the study. Cholesterol levels were not measured in the study and it should be mentioned that thrombolytic therapy was introduced in our hospital after the collection of the studied patients. Therefore, is not possible to generalize the results in a population of patients with stroke who undergo thrombolytic therapy. Although statin therapy may increase the risk of intracerebral haemorrhage, it has been shown that patients treated with statins before stroke had a better response to thrombolytic therapy [38]. On the other hand, in a study of gene expression of cerebral endothelial cells in rat brain tissue, atorvastatin reduced exogenous tPA-aggravated cerebral endothelial genes that mediate thrombosis and blood-brain barrier permeability. This could contribute to the beneficial effects of statins on thrombolytic treatment of acute stroke [39].

## Conclusions

The present findings indicate that pretreatment with statins, hypercholesterolemia or both in ischaemic stroke patients could have neuroprotective effects with reduced neurological deficits at presentation, lower early death and dependency rate, thus increasing the chances for good outcome. However, statin therapy may increase the risk of intracerebral haemorrhage, particularly in the setting of thrombolysis.

## List of abbreviations

ANOVA: Analysis of variance; CI: Confidence interval; COPD: Chronic obstructive pulmonary disease; CT: Computed tomography; MMP: Matrix metalloproteinase; MRI: Magnetic resonance imaging; mRS: Modified Rankin scale; OR: Odds ratio; SD: standard deviation.

## Competing interests

The authors declare that they have no competing interests.

## Authors' contributions

AA was the principal investigator, designed the study, diagnosed and took care of the patients, contributed to analyze the data, interpreted the results, wrote the paper, and prepared the final draft. He was also responsible for editorial decisions including the selection of the journal. LGE was the statistician, participated in the study design, analysis and interpretation of data and wrote the part of the paper related to the statistical analysis. MO, CT, MB and JM participated in the collection of data, medical care of the patients and review of the manuscript for intellectual content. All have read and approved the final draft.

## Pre-publication history

The pre-publication history for this paper can be accessed here:

http://www.biomedcentral.com/1471-2377/10/47/prepub
